# Common themes in tetrapod appendage regeneration: a cellular perspective

**DOI:** 10.1186/s13227-019-0124-7

**Published:** 2019-06-17

**Authors:** Bess M. Miller, Kimberly Johnson, Jessica L. Whited

**Affiliations:** Department of Stem Cell and Regenerative Biology, Harvard University, 7 Divinity Ave, Cambridge, MA 02138 USA

**Keywords:** Appendage, Regeneration, Tetrapod, Limb, Tail, Antler, Digit tip

## Abstract

Complete and perfect regeneration of appendages is a process that has fascinated and perplexed biologists for centuries. Some tetrapods possess amazing regenerative abilities, but the regenerative abilities of others are exceedingly limited. The reasons underlying these differences have largely remained mysterious. A great deal has been learned about the morphological events that accompany successful appendage regeneration, and a handful of experimental manipulations can be reliably applied to block the process. However, only in the last decade has the goal of attaining a thorough molecular and cellular biological understanding of appendage regeneration in tetrapods become within reach. Advances in molecular and genetic tools for interrogating these remarkable events are now allowing for unprecedented access to the fundamental biology at work in appendage regeneration in a variety of species. This information will be critical for integrating the large body of detailed observations from previous centuries with a modern understanding of how cells sense and respond to severe injury and loss of body parts. Understanding commonalities between regenerative modes across diverse species is likely to illuminate the most important aspects of complex tissue regeneration.

## Introduction

Regeneration is the replacement of lost parts with a perfect copy following injury. Many vertebrate species are capable of impressive feats of complete regeneration; salamanders are particularly gifted in this regard, as they are able to replace lost limbs, tails, parts of their brain, and more. In mammals, regeneration in which the structure of lost tissue is recapitulated appears to be limited to the distal tip of the digit. Although the liver grows new tissue following tissue loss, the original liver structure is not reformed in this process, and it is thus not considered true complete regeneration. Therefore, examining common themes in appendage regeneration offers a potentially unique platform to directly compare regenerative mechanisms across tetrapods, from amphibians to mammals, and to consider how common themes might be used to improve overall regenerative abilities in mammals.

Appendage regeneration is a complex process, which relies on multiple cell types to act in harmony. Recent advances allowing tracking and manipulation both of separate cell types and of individual cells have permitted a much finer view of the cellular basis of regeneration. This review will examine appendage regeneration in tetrapods with a specific focus on limb regeneration in salamanders, tail regeneration in lizards, antler regeneration in deer, and digit tip regeneration in mice to highlight common cellular mechanisms employed across regenerative modalities and consider potential reasons that may underlie the restriction of regenerative abilities in mammals. While wound healing is intimately connected to the complex events underlying regeneration, healing of wounds restricted to cutaneous tissue has been extensively reviewed elsewhere (e.g., [1–6]) and will not be covered per se in this review. Although deer antler replacement is not strictly speaking true regeneration, as it is prompted by seasonal changes in hormone levels rather than injury, it remains a rare case of complete replacement of a full appendage in mammals and will be instructive as to innate mechanisms of mammalian post-developmental appendage replacement.

## Overview of appendage regeneration and its limitations in tetrapods

Complete regeneration of complex body parts in vertebrates manifests in many lineages and structures. Generally, in vertebrates, regeneration is “unidirectional,” meaning that the missing piece is replaced by the body, but the piece itself does not regrow the rest of the organism. The most readily appreciated structures which undergo regeneration in tetrapods are appendages, for example, limbs, tails, and antlers. These structures can be lost to predation, autotomy, or seasonal shedding, after which they are regenerated with full or partial fidelity, depending on these species. Many examples of appendage regeneration are amenable to laboratory study following experimental amputation.

While many vertebrates have remarkable regenerative abilities, these abilities are not infinite, and they are subject to both spatial and temporal restrictions. In mice and humans, limb regeneration is naturally restricted to the distal-most portion of the digits ([7–10] and reviewed in [11]). Furthermore, human digit tip regeneration is anecdotally more successful in children and young adults, and it has only been documented in the medical literature up to age 13 [7]. Fetal mice possess the ability to regenerate a significantly larger fraction of the developing digit than their post-natal counterparts.

Frogs are more successful regenerators as tadpoles than they are as adults. As frogs undergo metamorphosis, they regenerate increasingly imperfect limbs culminating in either the regeneration of only a cartilage spike encased in skin or complete loss of regeneration, depending on the species of frog (reviewed in [12]). Imperfect regeneration is also a feature of lizard tails following autotomy, the spontaneous release of the appendage in response to predation (reviewed in [13]). Following regeneration of lizard tails, the vertebra is replaced with an unsegmented hollow cartilage section [14]. Dorsal root ganglia and gray matter of the spinal cord are not regenerated; rather, the regenerated tail is innervated by nerves extending from the proximal intact tissue [15–17]. Furthermore, the fracture planes seen in the intact tail, which permit autotomy at precise locations, are not regenerated (reviewed in [18]). Thus, lizards are distinctively better regenerators than other amniotes, but they have limited appendage regenerative abilities beyond this imperfect tail, and they do not regenerate limbs.

Many species of salamanders can replace entire limbs [19]. Axolotls are a popular neotenic salamander model for regenerative research that can be reared in the laboratory and readily regrow limbs, digits, jaw, tail, heart, gills, and liver upon experimentation. Even when experimentally forced to undergo metamorphosis by thyroxine administration, metamorphosed axolotls retain the ability to regenerate skin and limbs, albeit at a significantly reduced rate [20]. Metamorphosed axolotls (“paedomorphs”) tend to regenerate improperly patterned limbs more often than their neotenic counterparts [20]. The overall trend toward more restricted regenerative ability with organismal aging appears to exist but is currently poorly understood [21–23]. Future work will be required to determine if mature animals are lacking an essential component, cellular or otherwise, that juveniles have, or if adults have an increased repertoire of antagonistic components. These possibilities are not mutually exclusive.

One potential underexplored restriction is the possibility that even in strong regenerators, the regeneration program may not be repeatedly deployable with complete success. While still an underexplored topic, the majority of newt limbs reportedly either do not regenerate or regenerate with defects following as few as five amputations [24]. For axolotls, some evidence now exists that multiple regenerative events can be supported in juvenile animals provided they are rather closely spaced in time [25]; however, other evidence indicates that the regenerative program can ultimately be pushed to exhaustion by repeated sequential amputations performed over the course of a year [26]. In contrast, zebrafish have been documented to regenerate tails up to 27 times [27], though some permutations on the proximo-distal axis patterning can occur in this context [28]. While these examples involve experimental injury, in the wild, many deer naturally undergo seasonal antler shedding followed by complete regeneration (reviewed in [29]).

## Vertebrate appendage regeneration follows a common morphological pattern

**Fig. 1:**
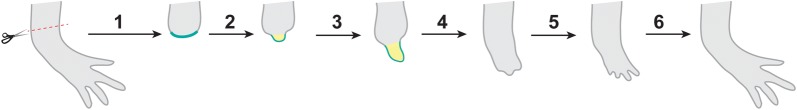
Salamander limb regeneration. (1) Following amputation, epidermal cells migrate over the amputation surface to create a wound epidermis (turquoise). (2) The formation of a blastema (yellow), a group of progenitor cells arising from dedifferentiation and stem cell recruitment, is cued. The blastema forms at the tip of the stump beneath the wound epidermis. (3) Progenitor cells in the blastema proliferate to expand the substrate pool for the new limb cells. (4) Blastema cells differentiate, tissues are patterned, growth continues (5) to form a perfect replica of the lost limb (6)

Appendage regeneration is a complex process requiring the regrowth of multiple tissue types in an organized pattern. In a general sense, all of the current models of vertebrate appendage regeneration undergo a stereotypical order of events to replace the lost tissue: epithelial closure of the wound site, dedifferentiation or activation of cells located at the injury plane to form a zone of proliferating cells at the amputation plane, and reformation of the lost appendage through progenitor cell proliferation, differentiation, and directed outgrowth (Fig. 1 for detailed illustration of this sequence in salamanders).

Initially, appendage loss results in the immediate formation of a blood clot at the wound site. Within the first 24 h in salamanders, epithelial cells adjacent to the wound site actively migrate over the wound to seal the amputation plane and form an epithelial layer termed the wound epidermis. Tissues within the underlying stump undergo histolysis, resulting in the degradation of collagen fibers and extracellular matrix proteins. Next, cells within the wound epidermis proliferate and thicken to produce the apical epithelial cap (AEC). Damaged nerve axons re-innervate the AEC, and the innervated AEC signals to the underlying stump cells to accumulate under the AEC. These migratory, activated progenitors consist of stem/progenitor cells and dedifferentiated cells, and collectively this enriched niche of cells is known as the blastema. Immune cells are present within the blastema, and actively contribute to blastema formation and regeneration, possibly through the release of cytokines [30]. Recently, an unexpected source of interleukin-8 (IL-8) cytokine, the blastema cells themselves, has been shown to promote immune cell infiltration at the site of limb regeneration, highlighting reciprocal relationships between these cell types in directing behaviors [31]. Ultimately, cells within the blastema likely undergo several rounds of amplification and then differentiate to replace musculature and skeletal elements. Following deposition of a new ECM, blood vessels reenter the blastema to facilitate and encourage blastemal cell survival during the redifferentiation phase. AEC maturation proceeds to generate a mature epithelium encasing the regenerated limb or digit tip.

## Early response to wounding and appendage loss

Following amputation, a cascade of cellular responses begins that ensures the animal does not hemorrhage to death and sets the stage for appendage regeneration. Clotting begins nearly immediately following amputation. While salamanders do not maintain a visible clot at the amputation plane, both lizard tails and mouse digit tips retain a clot at the amputation plane for about a week post-amputation; in mouse digit tips the presence of this clot is required for regeneration to occur [32, 33].

After the immediate wounding response, inflammation takes hold in the amputated limb or digit tip. The nature of inflammation in amputated appendages fated to regenerate is still hotly debated, and more experimentation will be necessary to tease out all of the effects. In axolotls, both canonical pro-inflammatory and anti-inflammatory cytokines have been reported to be upregulated in the early stages of regeneration [30]. Human digit tips are naturally more difficult to study post-amputation so information in this context is sparse, but recent data have suggested that exudate from fresh digit tip amputations is abundant in inflammatory cytokines IL-1*a*, IL-4, IL-6, and TNF-*a* [34].

## Formation of the wound epidermis is an integral early event in appendage regeneration

In salamanders, formation of the wound epidermis is absolutely required for limb regeneration. This early epidermis is histologically distinct from the mature epidermis covering the stump. It lacks glandular structures as well as the thick mat of collagen that lies beneath fully differentiated epidermis. The lack of collagen has been postulated to enable unfettered molecular communication between the wound epidermis cells and the cells at the tip of the stump (such as muscle, cartilage, bone, dermis, nerve) [35]. The wound epidermis maintains its collagen-devoid state well into the regenerative process, even after the formation of the progenitor-rich pool collectively called the “blastema.” These histological observations prompted experiments in salamanders whereby wound epidermis formation was physically prevented by inserting a partially skinned, amputated limb into the body cavity and allowing it to heal inside and regenerate if possible [36]. Wound epidermis formation can also be inhibited through repeated daily removal of the wound epidermis following ordinary amputation [37]. Both operations impeded regeneration. A later tactic was developed in which mature epidermis is immediately sutured across the raw stump following amputation [38]. While this procedure is not always successful, in the cases where it is, all outwardly observable features of limb regeneration fail to occur. Most notably, the blastema does not form, and limbs do not regenerate. In cases where the operation is mostly successful but some aspect of the suturing does not hold, a miniature blastema can form in that location ([38] and JW, personal observation). If wound epidermis is coaxed into an “eccentric” location, for instance, to the side of the blastema rather than atop it, in time the blastema cells will themselves re-localize to become positioned beneath the wound epidermis [39]. The wound epidermis expresses several Fgfs (Fgf1, Fgf2, Fgf8) [40–43], and administration of either Fgf1 or Fgf2 promotes blastema cell proliferation [44–47]. Collectively, these experiments show that wound epidermis is required for limb regeneration and that it may influence the formation of a blastema and/or the migration of blastema cells to their necessary location. However, much more experimentation will be necessary to determine the precise role of the wound epidermis in the overall process. It is possible, and perhaps likely considering some published reports, that some early steps of cellular activation following amputation are independent of the wound epidermis [48]. If so, the wound epidermis may be more important for sustaining cellular responses required for limb regeneration (for example, localized cellular proliferation) rather than initially instigating them.

Interestingly, a requirement for specialized epidermis is shared in human digit tip regeneration. Children who have experienced traumatic fingertip loss will not regenerate the fingertip even at ordinarily permissive proximal–distal levels if the open wound is sutured closed [7]. Mice also elaborate a specialized type of epidermis to cover the amputated digit stump, but the timing of wound epidermis growth is dramatically delayed compared to salamanders [49], and there is some evidence that speeding up the timing of wound closure in the mouse digit tip does not impede successful regeneration [49].

Following tail loss in lizards, keratinocytes migrate across the amputation plane and form a wound epidermis, which then continues to thicken, in a process that takes several days [16]. Dermis formation is delayed until later regenerative stages when cartilage, muscle, and adipose tissue in the regenerate are almost done differentiating [16]. The epidermis in lizard tail regeneration appears to play a similar role to the wound epidermis in salamander limb regeneration. It secretes proteases that facilitate degradation of mature stump tissues, as well as producing Wnt5a and Fgf2, which contribute to blastema cell proliferation and migration [50]. In mouse digits and lizard tails, fairly extensive osteoclast activity is required prior to wound epidermis formation to trim back the remaining digit or vertebral bone and facilitate epidermal closure of the wound site [16, 51].

In contrast, regeneration in deer antlers proceeds down a modified epithelial program as compared to other organisms. Epidermal cells from the pedicle rim similarly migrate underneath the scab formed following antler casting in order to re-epithelialize the wound area. However, rather than thickening and forming a structure akin to the AEC, dermis also forms underneath the epidermis, resulting in full thickness skin covering the wound surface [52, 53]. This is a notable difference, as covering amputated salamander limbs or mammalian digit tips with full thickness skin prevents limb regeneration. There is experimental evidence that direct interaction with the skin is not required for antler regeneration, as separating the skin from the pedicle bone with an impermeable membrane results in regeneration of skinless antlers [54]. Thus, it appears that the epidermal–mesenchymal signaling axis integral to other forms of appendage regeneration is dispensable for deer antler replacement.

## Role of the blastema in appendage regeneration

**Fig. 2:**
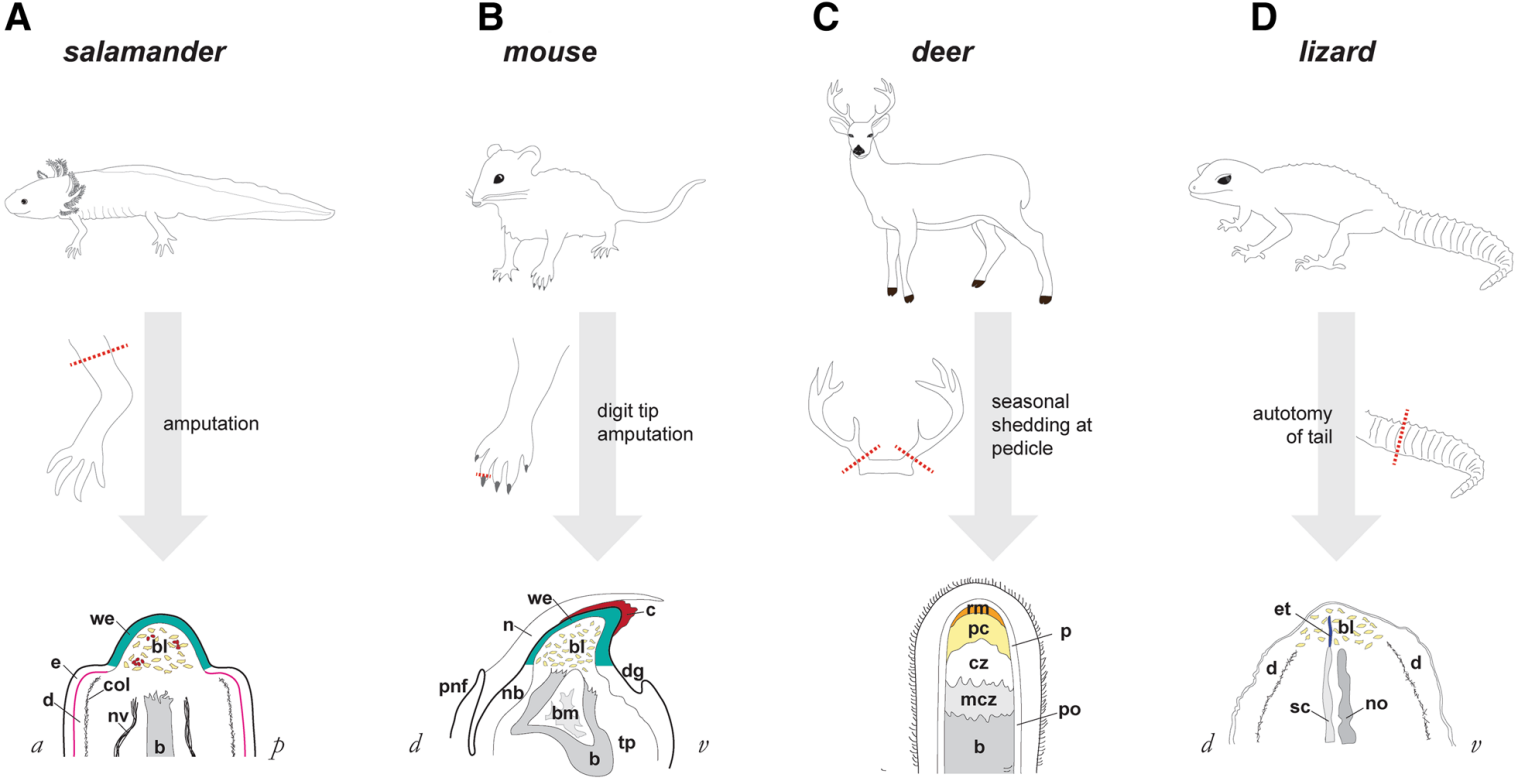
Comparative anatomy of regenerating appendages in salamander, mouse, deer, and lizard. **A** In axolotl, amputation at the mid-humerus level produces a mid-bud-sized blastema (bl, blue cells) within 7–23 days post-amputation, depending upon animal size. Intermingled with blastema cells are blood cells (shown in red). Overlying the blastema is wound epidermis, also known as apical epidermal cap. Note that more proximal epidermis (e) shows distinctly visible basal lamina (magenta), and dermis (d) is bound by a thick collagen mesh (black hatch marks). These features are absent beneath wound epidermis. Nerve: nv; bone: b. Adapted from Payzin-Dogru and Whited, 2018. **B** In mouse, amputation through the distal-most phalange at the level of the nail bed, produces a blastema (bl) growth at the distal tip beneath both a clot (c) and a wound epidermis (we), shown around day 10 post-amputation. The nail (n) has already grown past these structures by this time. Histolysing bone (b) is shown with bone marrow (bm). Nail bed: nb; toe pad: tp; proximal nail fold: pnf; distal groove: dg. Adapted from Lehoczky et al., 2011, Fernando et al., 2011, and Payzin-Dogru and Whited, 2018. **C** In deer, antlers are shed from pedicles. Regrowth occurs in zones distal to the bone (b). Mineralized cartilage zone: mcz; cartilage zone: cz; precartilage zone: pc; reserve mesenchyme: rm; periosteum: po; perichondrium: pc. Adapted from Kierdorf et al. [55]. **D** In lizards, autotomy of the tail produces a blastema-like structure (bl) encasing ependymal tube (et) at the tip of the stump spinal cord (sc). Notochord: no; dermis: d. Adapted from Gilbert et al. [13]

The blastema is a critically important structure for regeneration of limbs, tails, and antlers. All of these structures rely on this mound of cells that lies beneath the wound epidermis atop the stump (Fig. 2). While the active proliferation zone in antlers and tails may not bear complete resemblance to limb and digit tip blastemas morphologically, the concept of relatively undifferentiated progenitors at the tip holds true (reviewed in [55]). Single-cell RNA-sequencing of blastemas from various appendages will be informative as to the extent of the differences between the blastemas formed in these species. In general, activated progenitor cells within the blastema are proliferative during their time here, and the combined blastema/wound epidermis structure can be reasonably considered to be a transient niche in today’s scientific framework. What is especially remarkable about this niche is that the animal can develop it essentially as necessary and that the niche itself will, in time, resolve into differentiated tissues and disappear. Because mammals do not ordinarily respond to most types of amputations by growing a blastema, understanding how blastemas form and how the blastema/wound epidermis niche is orchestrated is of paramount importance to regenerative medicine.

## Progenitor activation to form the blastema

In appendage regeneration in salamanders and mice, degeneration of local tissue architectures in the stump, a process referred to as “histolysis,” precedes blastema formation. In salamanders, the activity of matrix metalloproteinases, initially secreted by the wound epidermis, is particularly important to this process [56, 57]. In lizard tail regeneration, keratinocytes migrating across the amputation or autotomy plane express MMP-9 to flatten the plane of tissue loss [33, 50]. Osteoclastic activity precedes antler casting, but antler casting is then followed by a small amount of bone formation to replace pedicle tissue lost with the antler rather than by histolysis [58–60].

Liberating progenitor cells to participate in regeneration is required, and a rather intuitive thought is that doing so necessarily requires the deconstruction of differentiated tissues that house cells in confined matrices. Muscle was one of the first tissues in salamander limbs observed to undergo histolysis by direct examination of tissue sections with light microscopy [61]. Later, proliferative muscle cells, labeled with tritiated thymidine, were observed to exist as increasingly fragmented and mononucleate the closer they were positioned to the amputation plane in regenerating *Ambystoma punctatum* larval limbs [62]. While not direct proof that descendants of these cells populate the regenerated muscle lineage, these studies underscore the relationship between localized architectural degradation and the early stages of limb regeneration in salamanders.

Activation of progenitor cells that are required to fuel the growth of the new limb tissue occurs simultaneously to histolysis. Here, we define activation as cell cycle reentry in response to environmental cues. Environmental cues may stem from the amputation event itself, from a structure-induced downstream of the amputation (such as the wound epidermis), or some combination therein. There exists a distinct possibility that progenitor cell activation does not require wound epidermis and even that some activation may occur in tissue distant to the amputation [48, 63]. However, in axolotls, the wound epidermis does contribute to progenitor proliferation and instructs the direction of migration, as discussed above. Transcriptomic analysis of 4 N cells during salamander wound healing and early blastema formation suggests that Wnt, Hippo, and TGF-β signaling are integral pathways during the progenitor activation stage of regeneration; and TGF-β has been experimentally shown to have an important transient role during the early stages of regeneration [31, 64]. TGF-β is also important during lizard tail regeneration [33, 65]. MARCKS-like protein is an extracellular factor that acts through unknown mechanisms and has been shown to promote cell cycle entry in regenerating newt limbs and unamputated axolotl tails—experimentally blocking its expression can diminish cell cycle entry in regenerating axolotl tails [66].

Live imaging of brainbow transgenic axolotl digit tips has shed light on the dynamics of cellular migration into the blastema [67]. While the digit tip does not contain muscle, this approach revealed diverse dynamics of migration and proliferation in connective tissue types. Although chondrocytes proliferated, they did not migrate to the amputation plane. In contrast, periskeletal cells and fibroblasts undertook multiple migratory waves into the blastema (a behavior likely reflecting their role as skeletal progenitors [68], discussed below), and fibroblast migration could be induced by platelet-derived growth factor signaling [67]. Similar studies are needed to better appreciate migratory dynamics during other models of appendage regeneration, but new methodologies that allow for real-time and in vivo cell tracking at a single-cell level should illuminate this important process.

## Progenitor cells can be derived from stem cells or dedifferentiation of mature cells

The source of activated progenitors that will give rise to the regenerated structures has been among the most intensively studied aspects of appendage regeneration. Activated progenitor cells may be either lineage-restricted or multipotent in their differentiation abilities, and may be derived from stem cell populations within the intact limb or from dedifferentiation of mature limb cells. Early lines of experimentation aimed at answering this question utilized animals whose tissues could be histologically distinguished (i.e., diploid vs. triploid animals) for grafting experiments [69–72]. More recently, GFP(+) donor tissues from transgenic axolotls were grafted into GFP(−) hosts, and because the expression of GFP was driven with a ubiquitous promoter, all descendants of the transplanted cells could be identified in the regenerate [73]. This experiment indicated that little transdifferentiation occurs between tissue types during regeneration. Indeed, other studies have also suggested that muscle is derived from lineage-restricted progenitors in salamanders. Interestingly, the identity of these progenitors depends on both life stage and species, as pre-metamorphic newts and axolotls depend on satellite cells for muscle regeneration, while post-metamorphic newts rely on dedifferentiation of mature myotubes [74, 75]. Similarly, mouse digit tip regeneration also obeys lineage boundaries from stump through regenerate tissue [32, 76]. However, in lizards, recent evidence suggests that there may be a degree of cellular plasticity between the cartilage and muscle lineages [77]. Skeletal elements in salamander limb regenerates appear not to arise from cartilage and bone in the stump, but rather from the adjacent periosteum tissue [68].

Recent single-cell RNA-sequencing studies also suggest plasticity within connective tissue and fibroblast populations during salamander limb regeneration, as reconstruction of blastema cell differentiation trajectories across regenerative time points indicates that fibroblasts can contribute to joint, cartilage, and bone in the regenerate [78]. Furthermore, fluorescent-based near-clonal labeling of cells in the intact limb provides additional evidence for multipotent lineage contribution by connective tissue populations during regeneration [79]. Whether fibroblast and connective tissue progenitors derive from rare specialized populations in the intact limb or from general activation of all fibroblast/connective tissue cells is still unclear. During deer antler regeneration, recent experiments suggest that blastema formation is dependent on resident stem cells within the pedicle as opposed to dedifferentiation, and these stem cells actively proliferate as wound closure is occurring, generating bone and cartilage in the regenerated antler [80]. The pedicle periosteum where these stem cells reside is also responsible for providing chemical and mechanical cues that stimulate growth of nerves, blood vessels, and skin in a lineage-restricted manner [81]. Single-cell RNA-sequencing of the antlerogenic periosteum suggested that only one major type of stem cell underlies initial antler development [82]; comparing these data to cells captured from the PP will be informative as to cellular differences underlying initial antler formation vs. antler replacement.

For all tissues and lineages, determining whether progenitors arise via dedifferentiation or via stem cell activity—or some combination therein—is important. This question must be answered for normally configured appendages as well as for appendages that have experimentally altered configurations such that expanded functions for cells might compensate for lack of typical progenitors. The latter consideration may be especially important for regenerative medicine.

## Progenitor cell survival in a hostile environment

A further important consideration for any type of tissue repair following traumatic injury is how to keep the progenitor cells, as well as necessary support cells, alive in a hostile environment. Traumatic injury often leads to the release of reactive oxygen species (ROS), which can exert oxidative stress and lead to cellular damage, but which are also necessary for several examples of tail regeneration [83, 84]. Cells at the site of injury may become quickly disconnected from their primary source of nourishment as vasculature regresses. Counteracting programs are likely to be required to spare important cells from death. Several studies have uncovered evidence of such counteractive forces operational during appendage regeneration. However, the relationship between cell death and regeneration is not simple, and in several contexts, components of the cell death pathway are essential for successful appendage regeneration. For example, Xenopus tadpoles cannot regenerate tails when programmed cell death is blocked via administration of a Caspase-3 inhibitor [85]. In the original report, this dependence was interpreted to indicate a possible need to remove some inhibitor cells, via programmed cell death, in order to enable regeneration. Several years later, a partial activation of a programmed cell death pathway was discovered to underlie the ability of muscle cells to participate in limb regeneration in newts [86]. The pathway is initially activated, but then aborted, and the engaged cells survive; the authors propose that cell death response activation may be a method to prompt dedifferentiation [86].

## Contribution of nerves to regeneration

Until the modern era of molecular genetics, researchers borrowed techniques from embryology to understand the basic framework of regeneration. As early as 1823, it was known that experimental denervation caused defects in—and sometimes even a complete failure of—regeneration in salamander limbs [87]. These observations were later complemented with parabiotic twin studies in salamanders that demonstrated that if limbs were forced to develop without innervation, these limbs could regenerate following amputation [88–90]. These data raise the question of why limbs ordinarily require nerves to regenerate, but limbs that develop without nerves are still capable of regeneration. One possibility is that in the innervated limb nerves take over the role previously played by a different tissue, but if the limb never becomes innervated this switch does not happen [91]. In salamanders, nerves are believed to be largely important for supporting blastema cell proliferation, although the precise mitogens secreted by nerves are still under investigation. Potential candidates include substance P, transferrin, nerve growth factor, anterior gradient protein, and neuregulin-1 [92–97]. Denervation of limbs simultaneously with amputation prevents blastema formation and leads to more extensive tissue histolysis; however, denervation at later stages once patterning has begun results in formation of a miniature limb that is otherwise patterned correctly ([87], reviewed in [98]).

In mice, there is conflicting evidence as to whether nerves are important for mesenchymal cell proliferation, as one study found no difference in cell turnover in denervated digit tips, while another found that loss of Schwann cells led to decreased blastema cell proliferation [99, 100]. Nerves are not absolutely required for mouse digit tip regeneration, but impaired innervation does lead to patterning defects in regenerated digit tips [99]. Nerve supply does not appear to be required for antler regeneration, although inputs from the nervous system may contribute to antler patterning as well [101, 102]. Thus, the contribution of nerves to complex tissue regeneration is variable between species, indicating that patterning information and mitogenic signals do not have irrevocably set cellular sources and rather are plastic across the evolutionary scale.

## A common role for macrophages?

The role of the immune system in regeneration is an area of active research. Differences in immune system components among mammals, amphibians, and reptiles will be discussed below; however, some common themes of immune cell utilization during regeneration are beginning to emerge across the evolutionary spectrum, specifically within the innate immune compartment. Recently, macrophages have been demonstrated to be required at early stages during axolotl limb regeneration, as their systemic depletion blocks outward blastema formation and downstream regenerative events [30]. Interestingly, this blockade is reversible, and if the macrophage lineage is allowed to replenish, these same limbs can be amputated at a more proximal level and successfully regenerate [30]. Future experimentation will be required to determine if the relevant macrophages are circulating or tissue-intrinsic. Macrophages are also required for mouse digit tip regeneration; clodronate-based depletion of macrophages inhibits bone histolysis and prevents blastema formation [103]. In salamanders, macrophages participate in clearance of senescent cells that accumulate following amputation [25]. Further investigation will be needed to determine whether this role is conserved in other models and to determine additional roles of macrophages in salamander limb regeneration. Current evidence suggests that adaptive immune cells are required at early stages of regeneration to clear the wound site of debris, and perhaps contribute to progenitor activation. The role of the adaptive immune system and necessary immune system inputs at later stages in regeneration remains to be fully understood.

## Systemic inputs in appendage regeneration

Early irradiation experiments in salamanders as well as more recent live imaging approaches demonstrated that local cellular inputs are sufficient for regeneration to occur [67, 104, 105]. However, systemic or long-range factors have also been implicated in appendage regeneration beyond the local structures of wound epidermis, nerves, and blastema cells, though decidedly less mechanistic information exists on systemic factors. For example, the thyroid gland, the pituitary gland, and the pancreas have all separately been demonstrated to be required for salamander limb regeneration [106–112]. Transcriptomic evidence suggests involvement of thyroid hormone signaling in lizard tail regeneration as well [113]. Antler replacement is of course dependent on seasonal variations in circulating levels of testosterone, although there is evidence that the effect of testosterone on antlers is indirect [114]. More evidence is needed as to the role of systemic factors in mouse digit tip regeneration. Interestingly, in mice, muscle injury on one side of the animal prompts muscle stem cells on the contralateral side to enter an alert state via a HGFA-mTOR signaling axis, demonstrating that in addition to systemic influences regulating a local injury response, injuries themselves result in system effects on other tissues [63, 115, 116]. Efforts aimed at understanding the interplay between local injuries and global responses will undoubtedly be a fascinating and important area of future investigation.

## Patterning and reconnection

Beyond just restoring the loss mass of tissue, true regeneration restores appendage patterning, size, and function to match what was lost. This is an amazing feat considering the animal may lose the appendage at any time in its life, and therefore, the regenerated appendage must be correctly calibrated to the current size of the animal. While there have emerged some fascinating connections between ion channels and achieving the correct size in zebrafish fin regeneration [117], in tetrapods, very few permutations on size, either naturally occurring or experimentally induced, have yet been shown [118, 119].

In addition to regrowing an appendage of the correct size, the regenerate also must recapitulate the structure of the lost tissue in order to be functional. In all the models discussed here, there remains much to learn about how the regenerated tissue knows which structures to make. In salamanders, blastemas are positionally autonomous shortly after forming [120, 121]. However, a distal blastema (one that will regenerate only a hand) can effectively be turned into a proximal blastema (one that regenerates hand and forearm for example) by application of retinoic acid [122]. As discussed above, nerves appear to influence patterning in mouse digit tip regeneration and deer antler replacement. In axolotls much positional information appears to derive from dermal fibroblasts [123], and connective tissue fibroblasts in mouse digit tips retain positional information as well [124]. Cellular inputs to positional information thus appear to vary to some degree evolutionarily, and it will be informative to understand the molecular signals provided by these various cell types and ask whether they are conserved across regenerative models.

The newly regenerated appendage must be wired to the stump vasculature and nervous system if it is to survive and to function. This process is largely under-studied and often presumed to rely on the redeployment of embryological mechanisms that may have drove the vessel precursors and the axons into the developing structure when it was first generated. However, this is likely a simplistic assumption even if many of the molecules are indeed reused. In the naïve, developing limb, the landscape that vessels and axons must traverse is likely different from the track they take out of a limb stump, through a sea of histolysis, and into a progenitor-rich field. In antler regeneration, axons may receive guidance cues from cells in the blood vessels, as axons that grow out from the pedicle are associated with the major blood vessels in the antler [125]. In amphibians, nerve growth factors appear to derive from blastema cells, as co-culture of blastema tissue and nerve cell bodies promotes axon regeneration [126]. Although revascularization of the regenerate is thought to proceed via angiogenesis from existing blood vessels in the stump, rather than via formation of new blood vessels [127] in salamanders, more work is needed to determine the factors specifically regulating this process.

## Evolutionary considerations

Perhaps the most salient question besides how these animals regenerate appendages is why some are so good at it, while others are not. Many hypotheses have been put forth to explain possible selective pressures acting upon traits required for appendage regeneration. Because many salamanders exhibit cannibalistic tendencies when housed together, a natural question is whether cannibalism has shaped the evolution of regenerative tendencies. Many salamanders mate a single time each year, usually once the last snow has melted and left behind ephemeral “vernal” pools of water, which can be quite small. A single clutch often contains hundreds of eggs, so a high concentration of hatchling salamanders is certainly possible several weeks later. Cannibalism among larval tiger salamanders has been studied in the laboratory, where some individuals within a group-housing setting are cued through unknown—possibly olfactory—mechanisms to develop specialized jaw structures that facilitate cannibalization, and this transformation is dependent upon animal density within the enclosure [128]. Because most salamanders do undergo the final stage of metamorphosis to become terrestrial, and even if they remain aquatic and creep along the bottom of a pond or lake, having functional limbs is essential for survival. Using this logic, one might anticipate that rodents should maintain or elaborate a regenerative program more substantial than they do. Hence, other explanations have been suggested to underlie the poor natural appendage regenerative abilities of mammals. The most common hypotheses posit a trade-off between regeneration and a program antagonistic to regeneration.

## Evolution of the immune system and regenerative abilities

One hypothesis concerning reduced regenerative abilities in mammals posits that a more sophisticated or active immune system in the mammalian lineage may hinder key steps in appendage regeneration, resulting in a scarring rather than regenerative response in mammals. It is interesting to note here that deer antler regeneration does not appear to be a completely scar-free process, as scarring of the pedicle surface following antler casting has been observed [60]. However, immune system maturation is accompanied by decreasing regenerative abilities in both mammalian skin and frog limbs (reviewed in [129, 130]). Although both innate and adaptive immune responses are present in salamanders and lizards, there are key differences between the mammalian, reptilian, and amphibian immune systems. For example, reptiles and amphibians lack lymph nodes and rely on circulating cells to achieve immune stimulation. Functional B and T cells are seen in reptiles and amphibians, but unlike B cells in mammals, B cells in amphibians and reptiles are phagocytic, suggesting they may have modified functionality [131–133]. Of particular interest, salamanders demonstrate a reduced pro-inflammatory response after amputation, in line with studies that demonstrate that the inflammatory environment of mammalian wound healing is detrimental to scar-free wound healing [134].

A corollary hypothesis is that during regeneration cells express regeneration-specific antigens and/or developmental-specific antigens, which are not recognized as self by the adaptive immune system, leading to destruction of cells that initiate pro-regenerative processes in mammals. Urodeles and anurans largely show only chronic rejection of allografts [135], rather than the acute rejection seen in mammals, suggesting a weaker adaptive immune response, which could contribute to a more permissive environment for regeneration.

## Relationship between cancer and regeneration

Cancer and regeneration share several obvious features, including high levels of cellular proliferation, altered differentiation states, cellular migration (in cases of metastases), need for blood vessel recruitment, and others. These shared features, combined with the observations that salamanders rarely exhibit natural tumors [136] and, in cases where tumors have arisen on limbs, bisecting them during amputation might cause some tumor cells to differentiate into normal limb tissues [137], have led decades-long speculation about the relationship between cancer and regeneration [138–140]. A common hypothesis is that an evolutionary trade-off occurred between mechanisms that protect against cancer (for instance, tumor suppressor activity) and mechanisms that promote regeneration [141]. However, the relationship is clearly more complicated, as highlighted even by the most well-studied tumor suppressor, p53. Intriguingly, the axolotl p53 gene sequence encodes a protein with many amino acids whose cognate sequences are among those linked to cancerous mutations in mammals, leading to the conclusion that axolotls “tolerate” these changes since they rarely get tumors [142]. p53 activity must first be downregulated to enable cell cycle reentry and blastema formation [143], but it is later required for limb regeneration [142, 143]; thus, even considering this single, tumor suppressor, activity is dynamically regulated and nuanced. Another example is *ARF*, a gene known to act as a mammalian tumor suppressor, but possibly absent from salamander genomes [144]. Inactivation of *ARF* and the *Rb* gene together enabled mammalian myocytes to reenter the cell cycle in culture, a defining feature of successful limb regeneration in salamanders [144]. There is also some evidence that highly regenerative animals can respond to cues that cause mammals to form tumors by instead mobilizing the regeneration program. Administration of coal tar, a carcinogen, to newt limbs rarely induced tumor formation. Instead animals most often grew an ectopic limb, some of which may have become innervated [145]. However, in other, later experiments, subcutaneous flank and tail administration of specific carcinogens often induced neoplasms, and sometimes these metastasized; later, evidence that some primary tumors and even some metastases regressed and possibly differentiated was collected [146]. In other contexts, such as administration of carcinogens to the blastema itself or nearby tissue, either rate of limb regeneration or morphology of the regenerated structure, or sometimes both were altered [147, 148]. These results indicate that in highly regenerative animals, perhaps cells actively engaged in regeneration interpret carcinogenic insults differently from those not [148], but more research is needed to draw this conclusion and to better understand the intersection of cancer and regeneration.

## Conclusion and outlook

There are clear similarities in overall strategy for appendage replacement across tetrapods at the cellular level. All successful regeneration events explored thus far require a mechanism for sealing the initial wound in a manner that favors future regeneration. Although mice, axolotls, and lizards form a wound epidermis that lacks underlying dermis and does not show scar formation following amputation, the skin covering the plane of antler loss in deer does form dermis. Therefore, a direct interaction between epidermis and underlying progenitor cells is not absolutely necessary for regeneration to occur in all systems. Progenitor cells are recruited from stump tissues, and this process largely occurs close to the plane of amputation or loss. Systemic hormonal influences do exist and can exert powerful controls on appendage regeneration. Innervation promotes regeneration and impairing it can lead to regenerative blocks or patterning defects. Necessary inputs from the immune system are an active area of investigation, and understanding these inputs will enhance our understanding of regenerative differences and similarities between species.

Similarities across classes of animals at a broad or outwardly observable level do not necessarily prove that the structures supporting appendage regeneration are orthologous structures. The molecular similarities and differences between blastemas and active zones of proliferation formed during regeneration of different appendages remain to be fully elucidated, and illuminating them is imperative to understanding shared versus divergent regenerative strategies across tetrapods, as well as developing blueprints to improve regenerative abilities in mammals. Application of new technologies such as single-cell RNA-sequencing and proteomics to this question should prove extremely informative for clarifying possible core mechanisms of appendage regeneration that can be interrogated functionally.
